# Middlesex Hospital Reception

**Published:** 1924-01

**Authors:** 


### RECEPTION AT THE MIDDLESEX HOSPITAL.

At a time like the present, when all the great London hospitals are appealing for money to enable them to continue their voluntary work, a reception such as that held at the Middlesex Hospital on Tuesday evening, December 18, is of peculiar interest, the research departments being open for inspection, while scientific demonstrations were given by members of the staff and medical school. The X-ray treatment designed to prevent the recurrence of cancer after operation has been brought at the Middlesex Hospital to a state of great excellence, and exhibitions were given of the methods of administration, which demonstrated the remarkable exactitude with which the treatment can be applied and the care which is taken to protect the nurses and doctors from any adverse effects upon themselves.

### The Investigation of Disease.

Guests were also shown how diseases are identified and investigated, and the laboratory diagnosis of tuberculosis. There were likewise insulin demonstrations showing its effects on normal and diabetic blood, and in another department were examples of colour photography and photographic investigation in cancer research. The analysis of water and milk were likewise explained, as were the preparation of microscopic slides and stains for microscopy. In the anatomy museum were stereoscopic photographs of the heart, examples of human jaws illustrating the different ages of man, and models of the developing eye. The Prince of Wales, who attended the reception, remarked that it was a revelation of the manifold activities of the Middlesex Hospital and congratulated the chairman and governors on having afforded the public an opportunity of judging for themselves the importance of its research work.

